# Chloroform Vapor in Ear-Ache

**Published:** 1860-08

**Authors:** 


Chloroform Vapor in Ear-Ache.—The use of oil and chloroform in ear-ache is familiar to many. A recent case treated by Dr. L. D. Ford, of this city, develops the use of chloroform vapor for this affection on a method which proved highly satisfactory. The patient, a child, suffered severely, and other remedies failing, Dr. Ford used the following extemporaneous expedient:

Taking a 2 oz. vial, a small opening was punched through the bottom, a little cotton-wool soaked with chloroform was then put into the vial. The mouth of the vial was now applied to the external meatus. The attendant then placing his lips to the punctured extremity of the vial, *blew the vapor into the external ear*. The relief was instantaneous, and the patient soon fell asleep, being cured by this single local application. We commend the above method as well worthy of trial in cases of this painful affection of childhood.—*Southern Med. and Sur. Journal.*

